# A hybrid method based on semi-supervised learning for relation extraction in Chinese EMRs

**DOI:** 10.1186/s12911-022-01908-4

**Published:** 2022-06-27

**Authors:** Chunming Yang, Dan Xiao, Yuanyuan Luo, Bo Li, Xujian Zhao, Hui Zhang

**Affiliations:** 1 School of Computer Science and Technology, Southwest University of Science and Technology, Mianyang, 621010 Sichuan China; 2 School of Science, Southwest University of Science and Technology, Mianyang, 621010 Sichuan China; 3 Sichuan Big Data and Intelligent Systems Engineering Technology Research Center, Mianyang, 621010 Sichuan China

**Keywords:** Semi-supervised learning, Relation extraction, Medical knowledge graphs, Residual network, Bootstrapping

## Abstract

**Background:**

Building a large-scale medical knowledge graphs needs to automatically extract the relations between entities from electronic medical records (EMRs) . The main challenges are the scarcity of available labeled corpus and the identification of complexity semantic relations in text of Chinese EMRs. A hybrid method based on semi-supervised learning is proposed to extract the medical entity relations from small-scale complex Chinese EMRs.

**Methods:**

The semantic features of sentences are extracted by a residual network and the long dependent information is captured by bidirectional gated recurrent unit. Then the attention mechanism is used to assign weights for the extracted features respectively, and the output of two attention mechanisms is integrated for relation prediction. We adjusted the training process with manually annotated small-scale relational corpus and bootstrapping semi-supervised learning algorithm, and continuously expanded the datasets during the training process.

**Results:**

We constructed a small corpus of Chinese EMRs relation extraction based on the EMR datasets released at the China Conference on Knowledge Graph and Semantic Computing. The experimental results show that the best F1-score of the proposed method on the overall relation categories reaches 89.78%, which is 13.07% higher than the baseline CNN.

## Background

Electronic medical records (EMRs) are digital information generated by medical staff using electronic systems, such as text, symbols, charts, data, and images [1]. Among them, unstructured texts (such as discharge summaries, medical records, surgical records, pathology reports, etc.) are a major part of EMRs, which are conducive to accurately describing the medical process. By identifying various named entities and the relations between them that are closely related to patients in EMRs, we can obtain valuable medical knowledge and patient health information [2]. For example, in “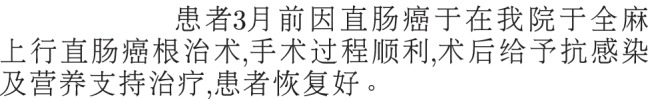 [The patient suffered from rectal cancer 3 months ago in our hospital under general anesthesia for radical resection of rectal cancer (DIXON), the operation process went smoothly, the postoperative anti-infection and nutritional support treatment was given, and the patient recovered well.]”, “
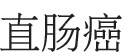
 [rectal cancer]” is a disease name, “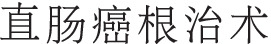 [DIXON]” is a treatment method (the two are called named entities in the relation extraction research), and the relation between them is “
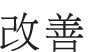
”, which belongs to the “TrAD” relationship in Table 3. We obtain a piece of medical knowledge that “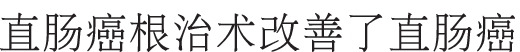 [DIXON improved rectal cancer]”. We can get a lot of such knowledge from EMRs and build a professional medical knowledge base, which is of great significance for promoting the establishment of a clinically-assisted decision system, personal health model, and intelligent medical question and answer(QA) [3].

Previous deep learning technologies have made important contributions in the field of relation extraction, but most studies only use a single convolutional neural network (CNN) or recurrent neural network (RNN) as a feature extractor, and few studies use deep networks to complete relation extraction in Chinese EMRs. Different from the news corpus commonly used in the open-domain field, Chinese EMRs have unique linguistic features, including a large number of long sentences and medical professional vocabularies. And the shallow neural network cannot well extract the complex semantic features in the text of EMRs. On the other hand, there is a lack of Chinese EMRs relation labeled corpus, and other language resources, so most of the supervised and distant supervised methods are not suitable.

Therefore, in this paper, we proposed a hybrid relation extraction method based on semi-supervised learning. This method combines the advantages of the deep residual network (ResNet) and gated recurrent unit(GRU) so that the model can fully learn the features of different levels and long-term dependency. Then we use the attention mechanism to further strengthen the key information. Secondly, and used the bootstrapping semi-supervised learning algorithm to adjust the training process. Experimental results show that our method can accurately extract relations in Chinese EMRs with only a small amount of labeled data, with the overall F1-score reaching 89.78%.

The rest of the paper is organized as follows: The background and related work is discussed in “Background” section. Then, in “Methods” section describes the detailed of our method. The datasets, the model parameters, and evaluation metrics settings are introduced in “Experimental settings” section. After that, the experimental results are shown in “Results” section. “Discussion” section is an analysis and discussion of the experimental results. Finally, brief conlusions are given in “Conclusions” section.

### Related Work

Relation extraction is essentially a classification task, i.e., classifying target entity pairs and sentences containing entity pairs according to the pre-defined relation categories. Table 1 shows the different classical methods for different stages of relation extraction. The previous relation extraction studies in open-domain filed mainly adopted feature engineering or kernel function method, which had poor classification performance, and required a lot of manpower to construct the feature set [4–6].

**Table 1:** Classical relation extraction methods

Classes	Principle	Classic Methods
Manual	Rule-based methods	Dependency parse trees[7]
Machine learning	Based on kernel functions	Convolution kernel[6]
	Based on feature vectors	SVM[4],CRF[5]
Deep learning	Based on convolutional neural networks	CNN[8, 9],PCNN[10],ResNet[2]
	Based on recurrent neural network	RNN[11],LSTM[12],GRU[13]
	Graph-based neural networks	GNN[14]

As the usage scenarios of deep learning become more and more extensive, many researchers apply neural network to relation extraction tasks. The commonly used models include CNN [8, 9], RNN [11] and its variant LSTM(long short-term memory) [12]. RNN can effectively learn the context dependence of text sequences, but it can not capture the features at the syntactic and semantic levels. CNN can capture the local information in the sentence, but ignores the role of global information. Zeng et al.[10] exploited piecewise convolutional neural networks (PCNNs) on the task of relation extraction and incorporated multi-instance learning to address the mislabeling problem. Lin et al.[15] proposed a CNN architecture with sentence-level selective attention for distant supervised relation extraction, which can make full use of all informative sentences and reduce the weights of those noisy instances. Considering the different contribution of every single pair of relational semantics in the sentence, researchers have introduced the attention mechanism, combined it with CNN and LSTM respectively, and achieved good results. Zhou et al.[16] combined bidirectional LSTM(BiLSTM) and multiple attention mechanisms for relation classification. Experimental results on the SemEval-2010 Task8 datasets show that this method outperforms most methods with only word vectors.

ResNet [17] is a new method for training very deep neural networks using identity mapping for shortcut connections. However, the effect of residual learning on noisy natural language processing tasks is still not well understood. Zhang et al.[2] proposed an attention-based ResNet to recognize medical concept relations in Chinese EMRs. The model achieved a F1-score of 77.80% on the manually annotated Chinese EMRs corpus and outperforms the state-of-the-art approaches. It shows that the residual network-based model can reduce the negative impact of corpus noise on parameter learning, and the combination of character position attention mechanisms will enhance the identification features of different entities.

GRU[18] is a commonly used gated RNN. Due to its relatively simple structure, GRU has a faster computing speed than LSTM. Moreover, due to fewer parameters, GRU has a better generalization effect on small sample data. The combination of GRU and other methods has also achieved good results in different fields. Hong et al.[13] adopted the method of relation extraction based on bidirection GRU (BiGRU) and attention mechanism (BiGRU-ATT) to retrieve these relations from Chinese medical text. The experimental results show that regarding Chinese medical entity relation extraction, they can achieve a better accuracy and recall than using a CNN.

In 2010, the i2b2/VA NLP challenge for clinical records proposed the medical entity relation extraction task with English EMRs, focused on assigning three relation categories that hold between medical problem, test, and treatment [19]. In recent years, the CCKS(China Conference on Knowledge Graph and Semantic Computing) has released Chinese EMRs named entity sharing tasks and annotated datasets since 2017, which has greatly promoted the research of Chinese medical information extraction. Fenia et al.[20] proposed an end-to-end method for the relation classification between drugs and drug-related entities. This method integrated neural network models such as BiLSTM, attention mechanism and transform, which could simultaneously extract the relations within and between sentences. Xu et al.[21] utilized a data-driven framework to extract structured records from the free-text narrative, with an F1-score of 84.6% on 24,817 Chinese EMRs datasets. Song et al.[14] used the graphical neural network (GNN) to generate high-quality dependent forests and solved the problem of low accuracy of dependent analysis in the biomedical relation extraction by taking dependent forests as external features. Liu et al.[22] proposed a capsule network model combining the shortest dependent path, and the F1-score of this model on the DDI Extraction 2013 datasets was 1.17% higher than that of the current best model.

Semi-Supervised Learning [23] uses a large number of unlabeled samples and a small number of labeled samples to train the classifier, which can solve the challenge of insufficient labeled samples. Semi-supervised learning has been successfully applied to many fields, such as marketing [24], security [25], etc. Semi-supervised learning is also widely used in the field of relation extraction, such as Zhang et al [26] proposed a semi-supervised biomedical relation extraction method that can effectively utilize unlabeled data to improve performance and reduce the reliance on labeled data.

CNN and RNN have always been the baseline models of relation extraction, and researchers have constantly innovated CNN and RNN. ResNet and BiGRU are widely used in relation extraction tasks. However, in the field of Chinese medicine, the feature extraction ability of them are still slightly insufficient to capture the complex semantic information in EMRs text. To solve the above problems, we propose a hybrid neural network relation extraction model based on ResNet, GRU and attention mechanism. Experiments show that our model achieves the best effect on our manually annotated Chinese EMRs corpus.

We make the following major contributions in this work:

- We propose a hybrid neural network model based on semi-supervised learning to extract relationships in Chinese EMRs.
- Our proposed hybrid neural network model achieves better results in performing semantic extraction, which cannot be achieved by other current models.
- The semi-supervised learning approach we take proves to be effective in expanding the data on a small annotated corpus.

**Fig. 1:**
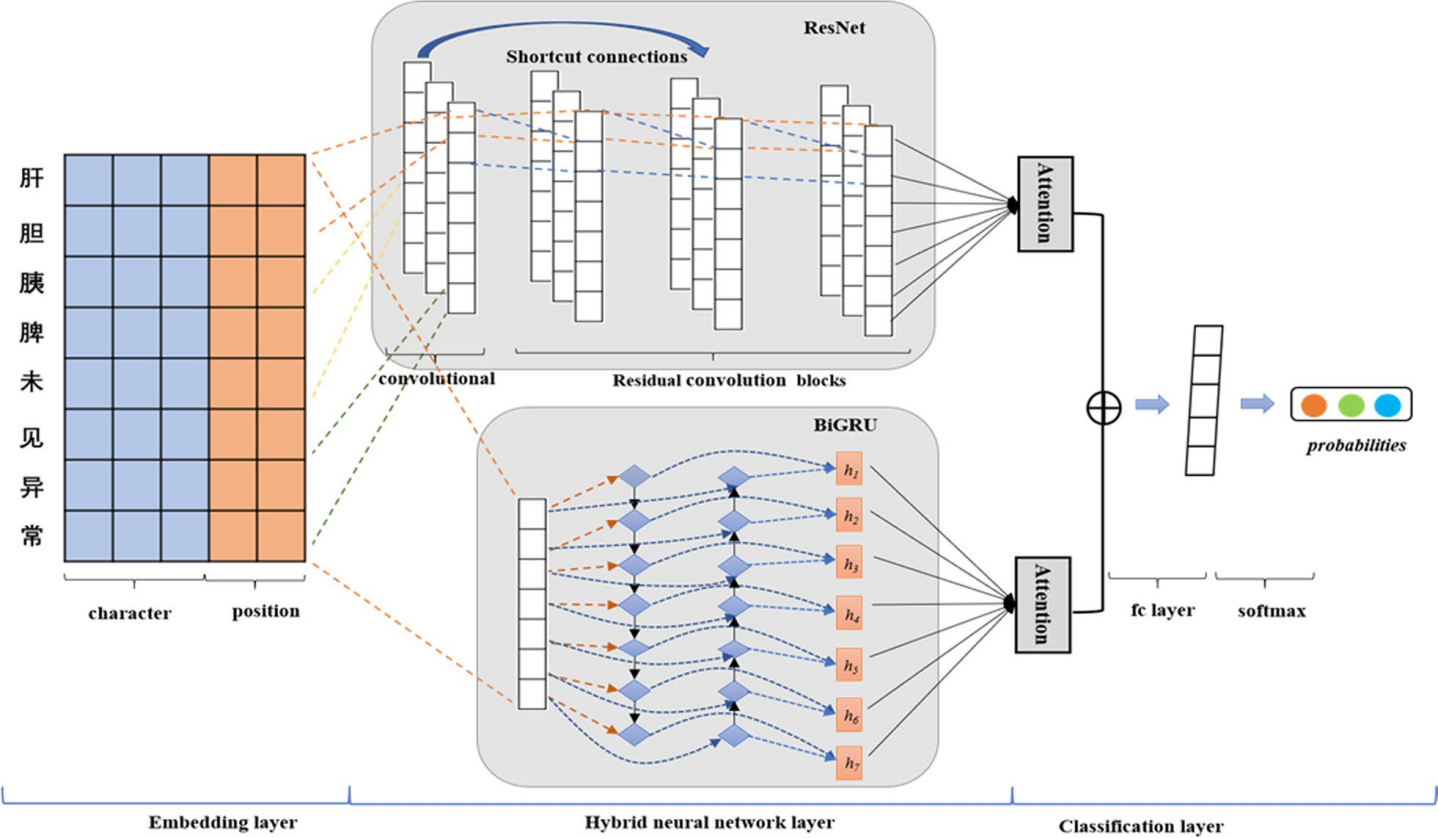
The architecture of the ResGRU-Att model

## Methods

### Relation extraction model

We propose the ResGRU-Att for relation extraction in Chinese EMRs. The model is composed of an embedding layer, a hybrid neural network layer (including ResNet, BiGRU, attention mechanism), and a classification layer. The overall architecture of our relation extraction model is shown in Fig. 1.

#### Embedding layer

For a given sentence $S=\{c_1,c_2,\ldots ,c_i,\ldots \}$, including the marked entity pair $e=[e_1,e_2]$. Each character $c_i$ in the sentence is mapped to a character embedding and two-position embedding. After the two vectors are spliced, The final vector representation of each character $x_i=[x_{iw}, x_{ip}]$ is obtained by splicing these two embedding vectors.

**Fig. 2:**
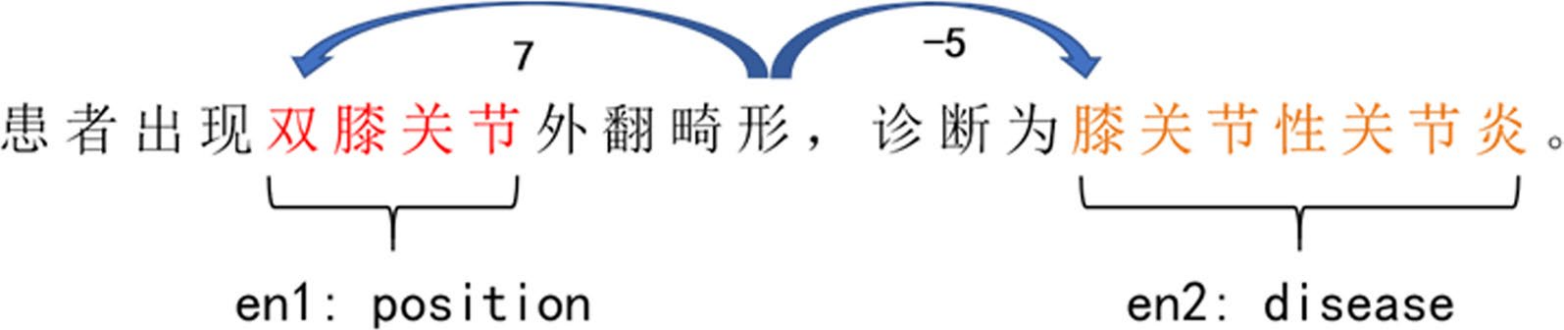
Example of position embedding

*character embedding* We use the *word*2*vec* tool to pre-train all medical record texts, and then use it to initialize the character embedding of the input sentence.

*position embedding* In the task of relation extraction, the words close to the target entities are usually informative to determine the relation between entities. Similar to Zeng et al.[27], position embedding reflects the positions of target entity pair and the relative distance between characters and the marked entity pair. Each character $c_i$ is mapped into two position embedding through random initialization. Figure 2 gives an example of the relative distance between a character and two entities, where the relative distances between “
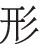
” and “
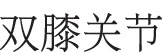
” and “
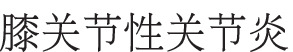
” are 7 and -5, respectively.

The input of model is the matrix *X*:

1 $$\begin{aligned} X=[x_1,x_2,...,x_i,...] \in R^{L\times d_v} \end{aligned}$$

Here, $x_i$ represents the vector representation of the $i_{th}$ character in sentence, and *L* is length. We set the dimension of character embedding to $d_w$ and the dimension of position embedding to $d_p$, and the vector dimension of the sentence is $d_v=d_w+2d_p$.

### Hybrid neural network layer

The hybrid neural network layer combines the features extracted by ResNet and BiGRU. While increasing the network depth, it ensures the model’s ability to learn local information and long-term dependency. Then the attention mechanism is used to further highlights significant information for relation classification.

#### ResNet

ResNet [28] consists of a standard convolutional neural network and four residual convolution blocks. Assuming that the vector matrix of consecutive *h* characters from the $i_{th}$ character in the sentence *S* is $x_{i:i+h-1}$, use the filter $W \in R^{ h\times d_v}$ to perform convolution operation on $x_{i:i+h-1}$ to obtain the feature $c_i$ in the window *h* as (2) shown:

2 $$\begin{aligned} c_i=f\left( w{\cdot x}_{i:i+h-1}\right) \end{aligned}$$

Here, *w* represents the weight parameter matrix of the filter, *b* is the bias term, and *f* is a nonlinear function.

**Fig. 3:**
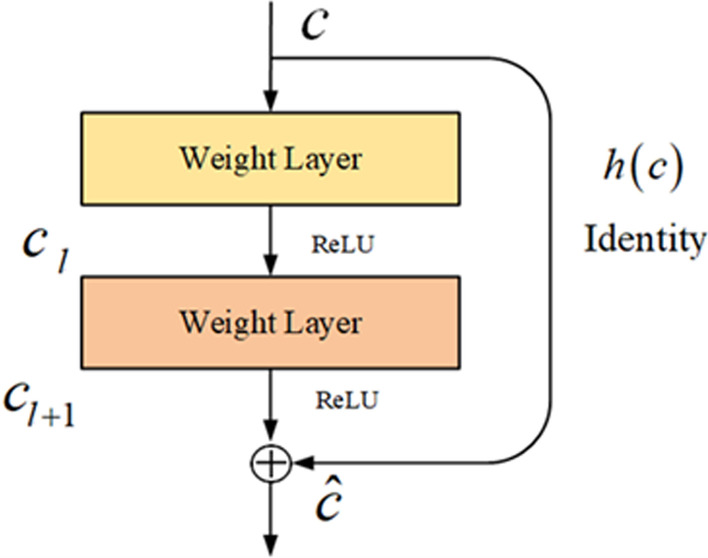
Residual convolution block

ResNet uses a shortcut connection based on the standard convolutional network, which connects the output characteristics of the underlying network to the high-level. Shortcut connection strengthens the multiplexing and transmission of features between different levels and avoids the problem of network degradation and gradient disappearance caused by too many layers. The structure of residual convolution block is shown in Fig. 3. Each block contains two convolutional layers. The ReLU function is used to activate the neuron after each convolutional layer. The features are directly passed to the next layer to realize shortcut connections between different residual convolution blocks by identity mapping.

Assuming that the input of residual convolution block is *c*, the output of block is expressed as:

3 $$\begin{aligned}&c_l=f\left( w_1\cdot c+b_1\right) \end{aligned}$$

4 $$\begin{aligned}&c_{l+1}=f\left( w_2\cdot c_l+b_2\right) \end{aligned}$$

5 $$\begin{aligned}&\hat{c}=g\left( c_l+h(c)\right) \end{aligned}$$

Here, $c_l$ and $c_{l+1}$ represent output of the first convolution and the second, *c* is output of the residual convolution block. $w_1,w_2 \in R^{h\times 1}$ are the weight parameter matrices of the two convolution filters. $b_1$ and $b_2$ are paranoid terms, *f* and *g* are activation functions. $h(c)=c$ is the identity mapping function, which is used to directly transfer the output features of the current layer to the next layer of the network.

#### BiGRU

**Fig. 4:**
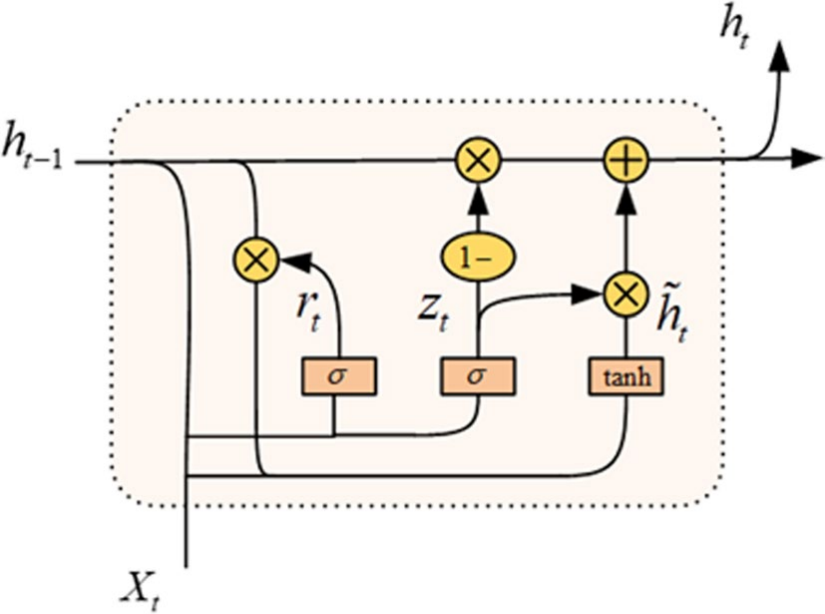
GRU unit

GRU is a variant of RNN that uses a gate structure to learn long-term dependent information, which can effectively solve the problems of gradient disappearance and explosion in RNN. Compared with LSTM, GRU has fewer training parameters and speeds up. The GRU unit structure is shown in Fig. 4.

Suppose the current time is *t*, $X_t$ is the character processed by the GRU unit at time *t*, $h_{t-1}$ is the hidden state at the previous time, and $h_t$ is the hidden state at the current time. The update method of the GRU unit is:

6 $$\begin{aligned}&z_t=\sigma (W_z[h_{t-1},X_t]+b_z) \end{aligned}$$

7 $$\begin{aligned}&r_t=\sigma (W_r[h_{t-1},X_t]+b_r) \end{aligned}$$

8 $$h_{t} = \tanh \left( {W_{h} \left[ {h_{{t - 1}} ,X_{t} } \right] + b_{h} } \right)$$

9 $$\begin{aligned}&(1-z_t){\otimes }h_{t-1}+z_t\otimes \widetilde{h_t} \end{aligned}$$

Here, $z_t$ and $r_t$ represent the update gate and the reset gate respectively. The update gate determines the information passed from the previous moment to the current, and the reset gate determines the information that was discarded in the hidden state at the previous moment. $h_t$ is the candidate’s hidden state. $W_z$, $W_r$ and $W_h$ represent weight parameters at time *t*, and $b_z$, $b_r$, and $b_h$ are bias vectors. $\sigma$ and $\tanh$ are activation functions. $\otimes$ is a matrix cross-product operation.

We use a BiGRU structure to calculate an input sequence at the same time, and stitch the two hidden states to obtain the final out $h_t=[\mathop {h_t}\limits ^{\rightarrow },\mathop {h_t}\limits ^{\leftarrow }]$.

#### Attention mechanism

In the actual relation extraction task, different characters are not equally important to judge the relationship type, and the decisive information may appear in any position of the sentence. The attention mechanism is introduced into the hybrid neural network layer to assign different weights to each character in the sentence, to emphasize the information that plays a key role in relation extraction and reduce the interference of other irrelevant information. The calculation formula is as follows:

10 $$a_{i} = Softmax(\tanh w \cdot H_{i} + b)$$

11 $$\begin{aligned}&S=\sum {a_i \cdot H_i} \end{aligned}$$

Where $H_i$ represents the input. $a_i$ is the attention weight given to the $i_{th}$ character in the sentence.

Firstly, the results of residual network and BiGRU are calculated respectively, then the two attention scores are fused to obtain the final output of the hybrid neural network layer:

12 $$\begin{aligned} S=(S_c \oplus S_G) \end{aligned}$$

Where $S_c$ is the attention score of the residual network, $S_G$ is the attention score of BiGRU, and *S* is the attention score of the hybrid neural network.

#### Classification layer

The final classification layer sends the features into a fully connected layer and a SoftMax classifier to complete the relation classification. The SoftMax classifier is an *r*-dimensional vector, where *r* is the number of relation categories, and the value of vector represents the probability of a relation category.

**Fig. 5:**
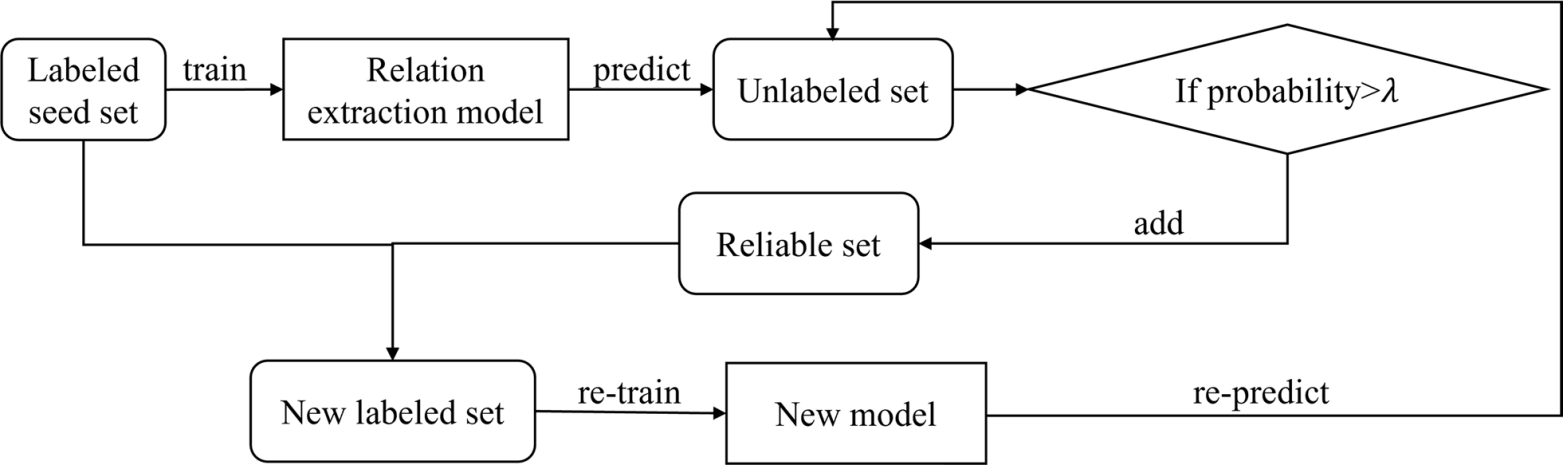
Training process of relation extraction model based on Bootstrapping algorithm

### Semi-supervised learning

To make use of the large amount of unlabeled data, a semi-supervised approach is the most appropriate. And we use the most widely used bootstrapping (see Table 2) method to learn. The basic idea is to use a small amount of seed datasets to select the highest score instance from unlabeled data, which significantly reduces the cost of manual labeling.

We use the bootstrapping to adjust the training process of relation extraction. The specific steps are:

**Table 2:** Bootstrapping algorithm

**Algorithm 1:** Bootstrapping	
**Require:** Labeled seed set L	
**Require:** Unlabeled set U	
**Require:** Reliable set N	
**Require:** Threshold	
** repeat:**	
Train a single relation extraction model on L	
Run the relation extraction model on U	
Find (at most) N instances in U that the probability predicted by the relation extraction model is greater than $\lambda$	
Add them into L	
**Until** No data points available in U	

As shown in Fig. 5, the bootstrapping is used to adjust the training process of relation extraction. The specific steps are as follows:

(1) First, use a small amount of manually labeled data as a seed set to train an initial relation extraction model (O-Relation).

(2) Use the O-Relation Model to predict the Unlabeled set, and output a relation label and a probability corresponding to the label for each piece of data. If the probability is greater than the threshold ($\lambda =0.7$), this data is divided into the reliable set.

(3) When the number of reliable sets reaches 1000, the seed set and the authentic set are merged into a new labeled datasets, and a new relation extraction model (B-relation) is re-trained.

(4) Repeat steps (2) and (3) until the unlabeled data set is cleared.

**Table 3:** Our relation annotation standard of the Chinese EMRs relation corpus

Entity pair category	Number of entity pairs	Relation category	Number of relations	Relation description
Disease-position	16538	DAP	304	Disease is applied to the position in the body
Symptom-position	19280	SAP	518	Symptom is applied to the position in body
		SNAP	893	Symptom is not applied to the position in the body
Test-disease	5743	TeRD	342	Test reveals the disease
Test-position	30673	TeAP	1194	Test is applied to the position in body
		TeCP	572	The results of the test contains the position in the body
Test-symptom	13617	TeRS	190	Test reveals the symptom
		TeAS	110	Test is applied to the symptom
Treatment-disease	5629	TrAD	679	Treatment is applied to the disease
		TrRD	227	Treatment (mainly surgery) reveals the disease
Treatment-position	8871	TrAP	128	Treatment is applied to the position in body

## Experimental settings

### Data collection and processing

In this paper, we built a small-scale Chinese EMRs relation corpus by manual tagging from CCKS in 2017 [29], 2019 [30], and 2020[31]. We established a Chinese EMR relation annotation standard according to Yang et al.[1] are shown in Table 3, which includes 7 categories of entity pairs and 11 relation. The entities are divided into the five categories of *treatment*, *disease*, *symptom*, *test*, and *position*. Our corpus has marked all entities and a small number of relations, so subsequent experiments do not need to perform named entity recognition tasks. In the end, we constructed contains 75,000 sentences, 37,000 entities and 7,000 entity relations.

**Fig. 6:**
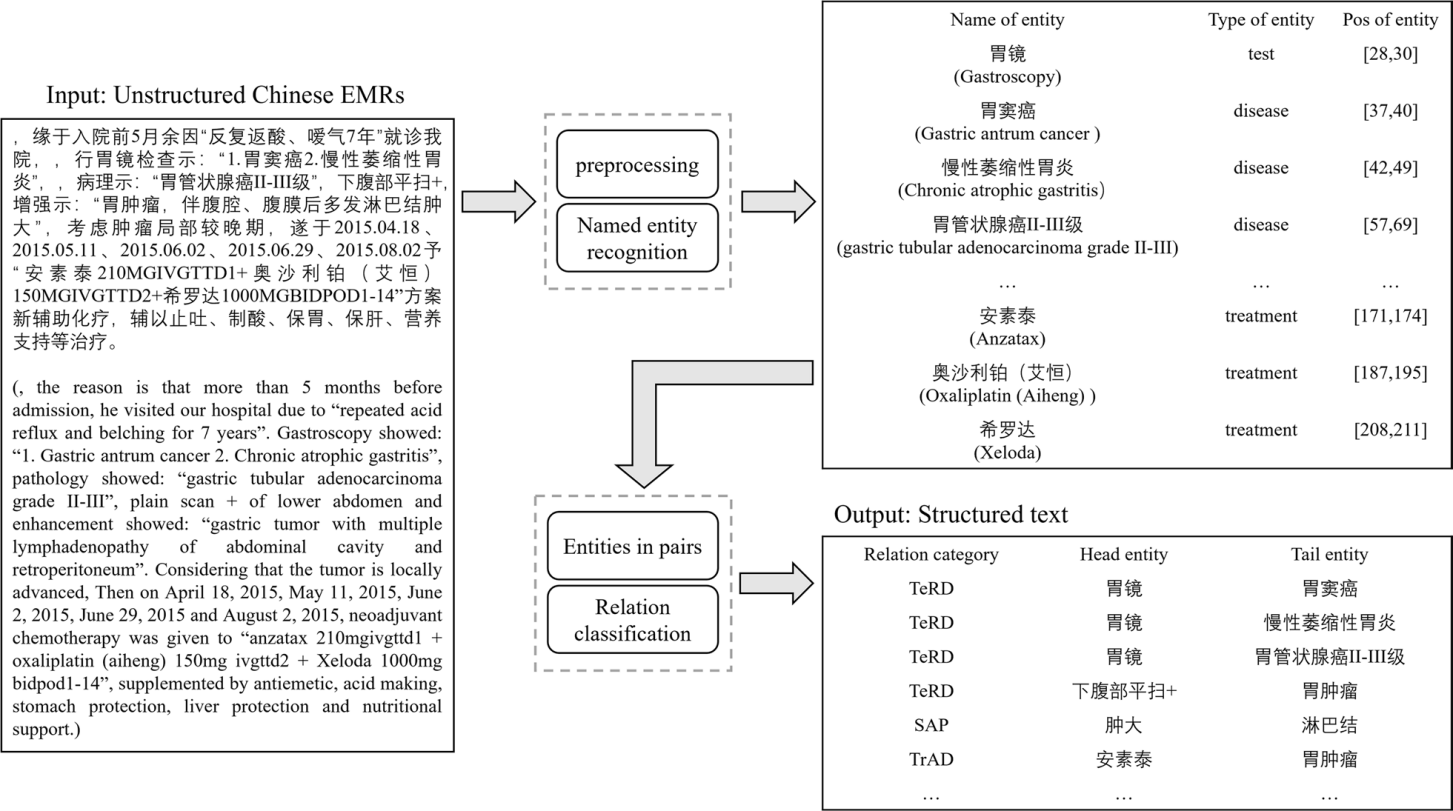
An example of Chinese EMRs relation extraction

Figure 6 shows an example of relation extraction. First, all the entities in a sentence (separated by periods) are grouped into pairs of entities according to their possible relationships. We train a classifier to predict which category of relationships the entity pair belongs to. If there is no relationship, it will be marked as “unknown”. We believe that “unknown” is a special relationship and will not be calculated in the final experiment. For example, there is indeed a relationship between “
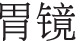
[gastroscopy]”(This is a test entity.) and “
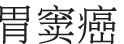
[gastric antrum cancer]” (This is a disease entity.), and the relationship between these two entities is “TeRD”( Test reveals the disease.). However, “Xeloda (treatment)” and “gastric antrum cancer (disease)”, for example, are not actually related to each other and will be marked as “Unknown”.

At the stage of bootstrapping label classification, 1000 labeled data would be generated in each iteration, so we divided the training set and test set in the ratio of 8:2 in the iteration stage for subsequent experiments. And if there is no special explanation, the subsequent experiment is to calculate the overall F1 value of the whole relationship.

### Models, parameters and evaluation metrics

We compared the ResGRU-Att with several neural network models and variants of the ResGRU described in the following.

CNN [27]: The CNN is baseline in whole experiment which contains a convolutional layer and a max-pooling layer.

CNN-Att: Based on the CNN, this model uses a character-level attention mechanism instead of the maximum pooling layer to aggregate the features.

PCNN [10]: The model divides one sentence into three pieces by the positions of two entities, and uses piece max-pooling to aggregate the features extracted from CNN.

ResNet [28]: The model consists of a convolutional network, four residual convolution blocks and a maximum pooling layer.

BiLSTM-Att/BiGRU-Att [16]: The two models use bidirectional LSTM and bidirectional GRU as feature extractors respectively, and then connect an attention mechanism.

ResGRU: This model is similar to our model, except that it does not use the attention mechanism.

The attention mechanism used in our model is the same as in CNN-Att, BiLSTM-Att, and BiGRU-Att.

We use precision, recall, and F1-score as the evaluation metrics of the experimental results. The experimental environment is set up as follows: CPU: Intel(R) Core(TM) i7-8700K CPU@3.70GHz, GPU: NVIDIA GeForce GTX 1080, OS: Ubuntu 18.04 LTS, RAM: 64GB, deep learning framework: Pytorch 1.2.0. Experimental parameters used in the relation extraction model are shown in Table 4.

**Table 4:** Experimental parameters settings

Parameters	value
Batch size	64
Dimension of character embedding	300
Dimension of position embedding	25
GRU hidden units	512
GRU hidden layer	3
Window size	3,5,7
Number of filters	128
Learning rate	0.015
Optimizer	Adam
Dropout	0.5

**Fig. 7:**
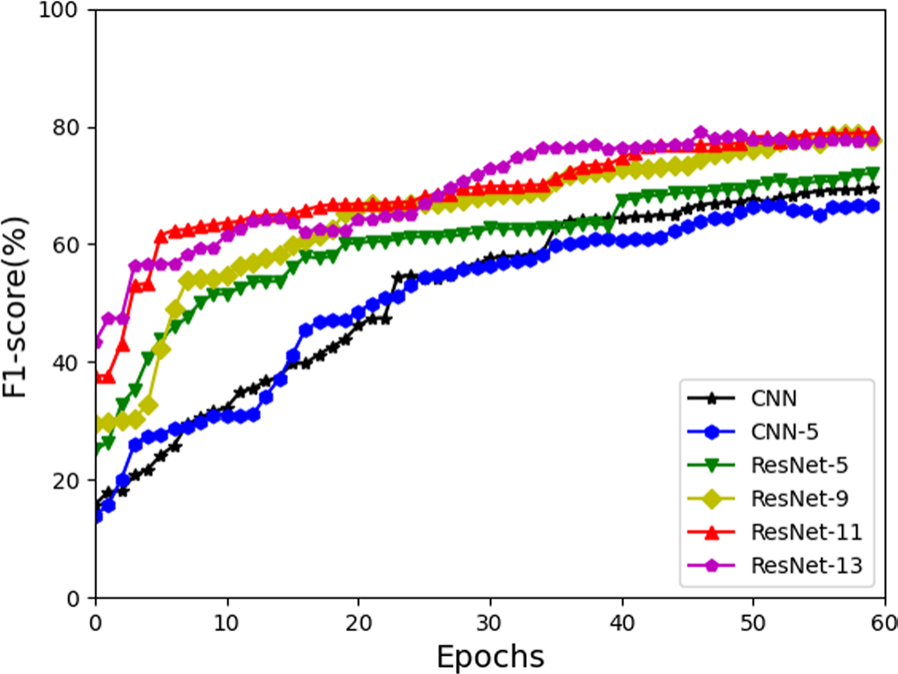
Comparison of F1-score of ResNet and CNN with different depths

## Results

### Comparison of ResNet and CNN with different depths

To explore the influence of network depths on the performance of relation extraction model, we compared CNN with single-layer, 5-layer and ResNet with 5-layer, 9-layer, 11-layer and 13-layer. This experiment was conducted in the training set and test set with a total data of 17000 and evaluated by calculating the overall F1 value of all relationships.

Figure 7 shows that CNN-5 is inferior to the baseline CNN, which indicates that directly increasing the depths of network layers on CNN is prone to over-fitting and performance degradation. The results of ResNet-9, ResNet-11, and ResNet-13 are significantly better than that of CNN and CNN-5. The results of ResNet-5 and CNN-5 are close, which shows that ResNet has little effect on shallow networks. As the depth of network increases, the performance of ResNet gradually increases, and the performance reaches saturation when the number of network layers is up to 11.

In the subsequent experiment, we use the 11-layer ResNet to reduce the amount of calculation and memory usage as much as possible, while ensuring that the model can achieve better performance.

**Table 5:** Comparison of F1-score of all models on different scale datasets

Models	$\hbox {N}=17000$	$\hbox {N}=30000$	$\hbox {N}=45000$	$\hbox {N}=60000$	$\hbox {N}=75000$
CNN	67.99	69.67	72.8	74.86	76.71
CNN-Att	70.49	73.57	75	78.44	81.06
PCNN	74.46	75.24	77.8	78.26	79.57
ResNet	78.27	79.75	81.52	83.34	86.13
BiLSTM-Att	76.12	80.98	82.71	84.96	85.21
BiGRU-Att	77.96	81.18	83.9	85.11	85.94
ResGRU	80	84.08	86	86.74	87.09
ResGRU-Att	**82.26**	**85.1**	**88.48**	**89.27**	**89.78**

Bold indicates the best value for this column

### Bootstrapping experiment

To verify the performance of bootstrapping algorithm, we verify the overall F1-score of multiple models under the increasing amount of data.

Table 5 shows the F1-score of all models on different scale datasets. It can be seen that as the datasets increases, the F-score of all models has been significantly improved. In the two training stages of the datasets increasing from $\hbox {N}=17000$ to $\hbox {N}=30000$ and from $\hbox {N}=30000$ to $\hbox {N}=45000$, the model performance improved the most. After the datasets increased to 60000, the model performance gradually became saturated, and the datasets stop training when it reaches 75000. Compared with the experimental results on the initial datasets, the F1-score of the CNN-Att increased by 10.57%, BiLSTM-Att increased by 9.09%, and the ResGRU-Att proposed in this paper increased by 7.52%. Except for the PCNN, the F1-score of all models has increased by more than 7%.

The ResGRU-Att has achieved the best results on both the initial and final datasets, and the F1-score has always remained above 80%. This shows that bootstrapping algorithm is suitable for expanding data. However, as the amount of data increase, bootstrapping still inevitably has the problem of semantic drift due to some ambiguous annotations, which is also a major disadvantage of bootstrapping. In terms of relation extraction, the learning effect of our model is better.

Since the model achieves the best effect when the amount of data is 75000, to ensure better performance of the model and reduce the amount of calculation and memory use, we will verify it when the amount of data is 75000 in subsequent experiments.

**Table 6:** Time comparison of all models on different scale datasets

Models	$\hbox {N}=17000$	$\hbox {N}=30000$	$\hbox {N}=45000$	$\hbox {N}=60000$	$\hbox {N}=75000$
CNN	13min43s	25min12s	40min5s	48min35s	57min3s
CNN-Att	17min18s	31min20s	47min35s	59min48s	1h20min15s
PCNN	13min20s	24min41s	41min48s	47min25s	56min25s
ResNet	50min35s	1h40min	2h39min11s	3h18min35s	4h25min24s
BiLSTM-Att	1h30min9s	2h38min41s	4h18min46s	5h6min29s	6h13min27s
BiGRU-Att	1h17min25s	2h30min22s	3h54min35s	4h55min4s	5h48min16s
ResGRU	1h50min46s	2h48min19s	5h1min8s	6h40min39s	8h14min8s
ResGRU-Att	2h4min5s	3h9s	5h24min34s	7h12min24s	8h51min21s

From Table 6, the difference in efficiency between CNN and PCNN is small. ResNet has increased the number of network layers compared to CNN, so there is a significant difference in the running time. CNN-Att has longer running time than CNN, but the difference is still small, and ResNet-Att has significantly more running time than ResNet by about 0.5h. BiGRU has a shorter running time than BiSTM.

**Fig. 8:**
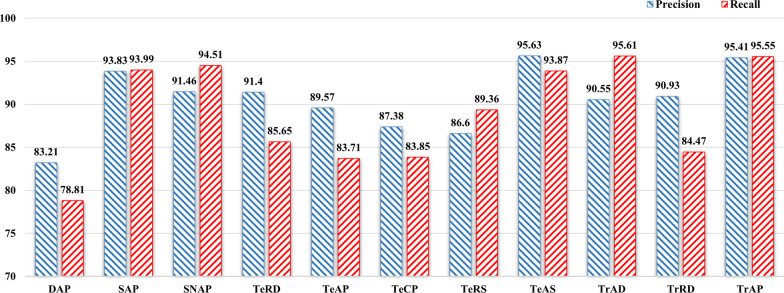
Comparison of precision and recall for the ResGRU-Att model on various relation categories

### Comparison with prior work

Figure 8 shows the accuracy and recall of the ResGRU-Att on specific relation categories. It can be found that ResGRU-Att achieves the highest accuracy rate on the category TrAS, reaching 95.63%, and the highest accuracy rate on the category TrAP, reaching 95.55%. Combining the two evaluation metrics of precision and recall, the ResGRU-Att performs best in the three relation categories between SAP, TeAS, and TrAP, with recall and precision exceeding 93%, and poor performance on DAP and TrRD, the precision and recall do not reach 85%.

**Table 7:** Comparison of performance for different models on overall relation categories

Models	Precision	Recall	F1-score
CNN	79.39	74.21	76.71
CNN-Att	85.46	77.09	81.06
PCNN	83.56	75.94	79.57
ResNet	88.44	83.94	86.13
BiLSTM-Att	85.4	85.02	85.21
BiGRU-Att	87.75	84.20	85.94
ResGRU	86.47	87.72	87.09
ResGRU-Att	**90.54**	**89.03**	**89.78**

Table 7 shows the comparison of different models on the overall relation categories. We can see that the ResGRU-Att has achieved the best experimental results, with the accuracy, recall and F1-score reaching 90.54%, 89.03% and 89.78% respectively. Compared with the baseline model, the F1-score increased by 13.07%. Compared with the BiGRU-Att, it has increased by 3.84%. and F1 increases by 3.65% compared with ResNet, which shows that the hybrid neural network combining ResNet and BiGRU has a better effect on feature extraction than ResNet and BiGRU alone. The ResGRU and the ResNet also perform well without using the attention mechanism, with the F1-score reaching 87.09% and 86.13% respectively. Which shows that the residual block in the residual network has a good ability to transmit information. The ResGRU that uses the hybrid neural network as the feature extractor performs better than the ResNet, and the F1-score is 0.96% higher. It shows that the hybrid structure of the gated cyclic network has stronger information retention ability. ResGRU-Att is 2.69% higher than ResGRU’s F1-score, indicating that the judgment of relationship types by different characters is very important, and the attention mechanism can further highlight the critical information of relationship classification, so our model also achieves the best effect.

**Table 8:** Comparison of F1-score for different models on various relation categories

Models	DAP	SAP	SNAP	TeRD	TeAP	TeCP	TeRS	TeAS	TrAD	TrRD	TrAP
CNN	64.86	78.04	75.95	79.51	85	74.29	80.4	66.76	83.9	72.74	82.36
CNN-Att	70.33	82.89	84.79	77.16	84.98	80.1	84.89	75.67	83.73	83.71	83.42
PCNN	69.53	84.74	80.06	75.69	82.28	76.36	79.95	75.43	84.74	83.44	83.05
ResNet	75.98	91.11	86.95	84.18	89.89	78.59	85.86	86	**94.99**	**87.62**	86.24
BiLSTM-Att	74.96	92.83	92.48	84.84	85.27	73.57	85.6	87.47	92.48	80.34	87.43
BiGRU-Att	75.34	90.62	90	87.43	**89.93**	83.18	85.27	81.84	92.08	82.24	87.44
ResGRU	78.53	92	**93.37**	**92.05**	85	83.39	84.46	80.13	91.83	87.11	90.17
ResGRU-Att	**80.95**	**93.91**	92.96	88.43	86.54	**85.58**	**87.96**	**94.74**	93.01	87.58	**95.48**

Bold indicates the best value for this column

Table 8 shows the comparison of F1-score of different models on specific relation categories. Due to previous experiments, we know that the number and distribution of entities are different, and some entities still have problems such as fuzzy boundary and nesting. The number of relationship categories, the distance between entity pairs and the complexity of sentence semantics lead to dissimilar effects of the model on different relationship categories.

Combined with the overall results, most models perform best on SAP and TrAD, but poorly on DAP. The ResGRU-Att has achieved better results than other models in nine categories of relation, and the F1-score on all relation exceed 80%, with the F1-score on SAP, SNAP, TeAS, TrAD and TrAP reaching 93.91%, 92.96%, 94.74%, 93.01% and 95.48% respectively. The best results on the other two relation categories of TeAP and SNAP are obtained by the BiGRU and the ResGRU, with the F1-score reaching 89.93% and 93.37%. Compared with the baseline CNN, the ResGRU-Att has the greatest improvement on the four relation categories of DAP, SAP, SNAP, and TeAS, with F1-score increased by 16.09%, 15.87%, 17.01% and 7.56%.

## Discussion

It is clear that the experiment takes longer as the number of network layers increases. As the amount of data increases, the longer the experiment takes.

From Figure 7, it is found that the ResNet series works better than the CNN series. This is because ResNet utilizes the shortcut connections between network layers to better integrate shallow and deep features, and improve the generalization ability of the model.

From Table 5, the ResNet, ResGRU and ResGRU-Att of the residual network are used to obtain better results than a single CNN and RNN. The reason is that the deep ResNet has a stronger feature extraction ability than the shallow network, which can avoid the overfitting problem of the baseline CNN. Why PCNN performance does not improve much when the amount of data increases? PCNN is an improved CNN to solve the problems of labeling errors in relation extraction using remote supervision and noise in feature extraction [10]. PCNN is much better than CNN when the amount of data is only 17000. Because PCNN is trying to avoid this problem, but the increasing data hinders the model learning, the model effect of PCNN is the worst.

From Table 6, since the structure of BiGRU is simplified compared to BiLSTM, the running time is shorter. ResNet-Att has a lot of improvements in network structure and more complex model than a single CNN or ResNet. Although it greatly improves the accuracy of extraction, it does require a greater time cost and a higher hardware configuration for the machine.

Also we can find that the bootstrapping semi-supervised algorithm is suitable for expanding the Chinese EMRs relation corpus, and the recognition accuracy on various relation categories has been significantly improved.

From Fig. 8, why does the same model perform differently in different relationship categories? We find that the symptom entities and test entities are densely distributed in the data set, and the composition structure is relatively single. The number of disease and treatment entities is small, the structure is complex, the boundary is fuzzy and the entities are nested. For example, the entity “
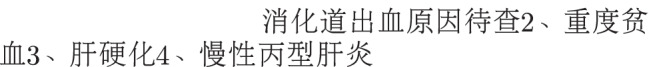
[the cause of gastrointestinal bleeding remains to be investigated 2, severe anemia 3, liver cirrhosis 4, chronic hepatitis C]” is wrongly identified as “gastrointestinal bleeding”, “severe anemia” and other entities. On the other hand, position-related entities often appear in the interior of treatment and disease. These three types of entities are prone to entity type ambiguity in the process of recognition. For example, the “
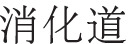
[digestive tract]” in the example above is actually a position entity, but the whole large entity is actually a disease.

When extracting relations, the above situation affects the effect of relation extraction and also makes the same model appear dissimilar in different relations. This difference is related to the number, length and composition of the entities themselves.

The Friedman test on Table 7 revealed that $p=0.0089$, $p<0.05$, indicating that these eight models differ significantly in three evaluation metrics and ResGRU-Att is significantly better than the other models. The ResGRU-Att with attention mechanism is better than the ResGRU, which shows that the judgment of relationship types by different characters is very important, and the attention mechanism can improve the performance of model.

From Table 8, ResGRU is 3.62% higher than ResGRU-Att on TeRD, which is due to the fact that the total TeRD entity pairs are the least, but there are relatively more relationships, and adding the attention mechanism instead leads to overfitting, making the effect less effective. BiGRU-Att is 3.39% higher than ResGRU-Att on TeAP, which is due to the fact that TeAP has the most relationships, and the separate BiGRU can reduce the risk of overfitting. Overall, the ResGRU-Att model proposed in this paper combines the advantages of ResNet and GRU neural networks and performs well in the overall relation categories.

The ResGRU-Att model shows differences in the extraction results of different relation categories. The reason may be related to the number of different relation categories, the distance between entity pairs, and the complexity of sentence semantics. And also related to the characteristics of the different models. Secondly, while using the bootstrapping algorithm to expand the training set, some relation categories introduce more noise, which will cause certain interference to relation extraction.

## Conclusions

In this study, we introduce a hybrid neural network method based on semi-supervised learning to extract entity relations from Chinese EMRs. This method firstly uses a residual network to reduce information loss during feature transmission and combines bidirectional GRU to capture long-term dependency and attention mechanisms to highlight key information. We train with a small amount of relation datasets annotated manually and use the bootstrapping algorithm to continuously expand the datasets. F1-score of our model exceeds 90% on five of the pre-defined relation categories and reaches 89.78% on the overall relations. Experimental results show that our method is suitable for extracting the relations between medical entities in Chinese EMRs. In the future study, we will attempt to add additional features and use the joint model or pre-trained language model to further improve the performance of relation extraction model.

## Data Availability

The data and code that support the findings of this study are available from https://github.com/yangchunm/Relation-Extaction
